# Development of Self-Assembled Nanoribbon Bound Peptide-Polyaniline Composite Scaffolds and Their Interactions with Neural Cortical Cells

**DOI:** 10.3390/bioengineering5010006

**Published:** 2018-01-13

**Authors:** Andrew M. Smith, Harrison T. Pajovich, Ipsita A. Banerjee

**Affiliations:** Department of Chemistry, Fordham University, 441 East Fordham Road, Bronx, New York, NY 10458, USA; asmith169@fordham.edu (A.M.S.); hpajovich@fordham.edu (H.T.P.)

**Keywords:** self-assembly, templates, tissue regeneration, peptide amphiphiles

## Abstract

Degenerative neurological disorders and traumatic brain injuries cause significant damage to quality of life and often impact survival. As a result, novel treatments are necessary that can allow for the regeneration of neural tissue. In this work, a new biomimetic scaffold was designed with potential for applications in neural tissue regeneration. To develop the scaffold, we first prepared a new bolaamphiphile that was capable of undergoing self-assembly into nanoribbons at pH 7. Those nanoribbons were then utilized as templates for conjugation with specific proteins known to play a critical role in neural tissue growth. The template (Ile-TMG-Ile) was prepared by conjugating tetramethyleneglutaric acid with isoleucine and the ability of the bolaamphiphile to self-assemble was probed at a pH range of 4 through 9. The nanoribbons formed under neutral conditions were then functionalized step-wise with the basement membrane protein laminin, the neurotropic factor artemin and Type IV collagen. The conductive polymer polyaniline (PANI) was then incorporated through electrostatic and π–π stacking interactions to the scaffold to impart electrical properties. Distinct morphology changes were observed upon conjugation with each layer, which was also accompanied by an increase in Young’s Modulus as well as surface roughness. The Young’s Modulus of the dried PANI-bound biocomposite scaffolds was found to be 5.5 GPa, indicating the mechanical strength of the scaffold. Thermal phase changes studied indicated broad endothermic peaks upon incorporation of the proteins which were diminished upon binding with PANI. The scaffolds also exhibited in vitro biodegradable behavior over a period of three weeks. Furthermore, we observed cell proliferation and short neurite outgrowths in the presence of rat neural cortical cells, confirming that the scaffolds may be applicable in neural tissue regeneration. The electrochemical properties of the scaffolds were also studied by generating I-V curves by conducting cyclic voltammetry. Thus, we have developed a new biomimetic composite scaffold that may have potential applications in neural tissue regeneration.

## 1. Introduction

The nervous system consists of a network of interconnected cells that play a critical role in the reception and transmission of electrical signals throughout the body [1,2]. However, damage to the nervous system caused by brain injuries or neurodegenerative disorders such as, Alzheimer’s, Parkinson’s, epilepsy, multiple sclerosis, or chronic traumatic encephalopathy, can lead to severe impairment in daily function and quality of life [3,4]. The slow growth and fragility of nervous tissue poses a unique challenge for treatment interventions. Current treatments are limited to nerve autographing and the use of nerve conduits [5], as well as development of novel antagonists [6]. These methods are challenged by the lack of donors, tissue rejection, scar tissue growth, implantation decay and lack of sufficient structural and biochemical information at the biomolecular level [7]. Tissue Engineering (TE) poses an alternative treatment option to conventional methods. TE seeks to repair, restore and replace damaged tissues and harbor growth of healthy tissue [8]. This is accomplished by creating a biomimetic three-dimensional matrix that exemplifies properties of the extracellular matrix (ECM), which can eventually aid in re-growing tissue [9]. These scaffolds are tailored to specific tissues to ensure compatibility and alleviate immune response and scar tissue growth and support new tissue by proper adhesion and integration [10,11]. 

Since the inception of TE, a multitude of materials, both natural and synthetic, have been discovered to promote neural tissue growth [12]. For example, functionalized carbon nanotubes and graphene nanotubes have been successful in promoting cell differentiation and migration, while efficiently maintaining conductive properties within the tissue [13,14,15]. Polymers [16] such as polyethylene glycol (PEG), poly ε-caprolactone (PCL), poly(lactic-co-glycolic acid) (PLGA) and poly-lactic acid (PLA) are some of the most widely used synthetic polymers [17]. Specifically, they have been used to create neural guidance conduits and cylindrical porous electrospun composites to promote axonal growth and to bridge neural ending defects [18]. For instance, it was reported that composites of poly ε-caprolactone electrospun membranes and gelatin improved cell adhesion, proliferation and differentiation of PC-12 nerve cells and supported neurite outgrowth [19]. In a recent study, it was shown that irradiation of graphitic carbon nitride integrated with graphene oxide (GO) that was bound to electrospun PCL/gelatin fibers resulted in neural stimulation upon irradiation with visible-light and thereby supported neuronal differentiation [20]. Amongst the naturally occurring proteoglycans, hyaluronic acid, chitosan, chondrotin sulfate and heparin sulfate have gained significant prominence [21] in the preparation of composite materials for neural TE. For example, in one study, chondroitin-6-sulfate and neural growth factor (NGF) were fused into PEG gels and promoted neurite extension and viability of cortical cells [22,23]. In a separate study, heparin-mimicking polymers—prepared by combining glucosamine-like 2-methacrylamido glucopyranose monomers with three separate sulfonated units—showed higher cytocompatibility and promoted differentiation of embryonic stem cells to neuronal cells as compared to natural heparin [24]. Forsythe and co-workers developed three-dimensional graphene-heparin-poly-l-lysine polyelectrolytes that promoted neuron cell adhesion, proliferation and neurite outgrowth [25]. It has also been shown that hyaluronic acid bound electrospun PCL scaffolds as well as agarose-chitosan blends enhance mechanical properties and increased proliferation of neural cells [26,27]. 

In addition to the aforementioned biomaterials, peptide amphiphiles have gained prominence in numerous biomedical applications due to their facile self-assembling properties, biocompatibility and relative ease of functionalization [28]. For example, when peptide nanofibers formed by self-assembly of amphiphilic (palmitoyl-GGGAAAKRK) were utilized for siRNA delivery into the brain, they showed higher intra-cellular uptake after being delivered intra-cranially [29]. Stupp and co-workers recently showed that hybrid DNA-peptide nanotubes that had been prepared by altering the sequence of the DNA strands and incorporating the cell-adhesion motif RGDS displayed selectivity and enhanced cell adhesion and differentiation of neural stem cells into neurons but not astrocytes [30]. Scaffolds formed by utilizing the self-assembling peptide RADA16-I have demonstrated potential in closing neural gaps and regenerating axons and healing spinal cord injuries [31]. Researchers have also developed hybrid matrices by combining Type I collagen and peptide amphiphile based nanofibrous scaffolds functionalized with IKVAV or YIGSR that showed specific responses to cerebellar cortex Granule cells and Purkinje cells. Specifically, the IKVAV hybrid scaffolds showed an increase in granule cell density and growth of Purkinje cell dendrite and axons in the presence of peptide nanofibers over specific concentration ranges compared to collagen [32].

In this work, we have developed a new biomimetic scaffold with potential for neural tissue engineering. We conjugated 3,3-tetramethylene glutaric acid (TMG) with isoleucine (Ile) to form a new bolaamphiphile, wherein TMG was the inner head group while the two isoleucine groups formed the tail groups at each end. The self-assembling ability of the Ile-TMG-Ile conjugate was probed at a pH range of 4–9. We observed that under neutral conditions, the conjugate self-assembled into nanoribbons, which were then utilized as templates for developing the scaffold. TMG has been shown to be biocompatible and is a well-known aldose-reductase inhibitor in vitro. It was once touted for its potential in inhibiting diabetic angiopathy and cataract formation by preventing the formation of sorbitol [33]. However, it was found to be relatively inactive as an aldose-reductase inhibitor in vivo, as significant amounts of TMG were unable to reach the retina or lens. We utilized TMG due to its unique structure containing the glutaric acid back bone functionalized with a cyclopentyl ring system. We conjugated it with isoleucine, as it is a key factor known to enhance the activity of alanine-serine-cysteine transporter (Asc-1), which mediates the release of Gly and Ser from neurons and modulates *N*-methyl-D-aspartate receptor (NMDAR) synaptic activity [34]. The nanoribbons formed upon self-assembly were utilized as templates for preparing tailored scaffolds for neural tissue regeneration. The template Ile-TMG-Ile nanoribbons were first conjugated with laminin, a major component of the extracellular matrix of vascular tissue in the brain. Laminin has been shown to increase the binding abilities of nanoscaffolds as well as to promote cell migration in newly formed cells [35,36]. A recent study conducted using a laminin functionalized PCl-chitosan scaffold showed increased mechanical properties, cell attachment and proliferation [37]. Another study found that laminin based scaffolds vastly improved neuronal survival in the injured brains of mice, which led to greater performance on spatial learning tasks [38]. 

We then conjugated the laminin bound assemblies with Artemin, which is a glial cell line derived neurotropic factor. Artemin is known to support signaling and increase growth in both peripheral and central nervous tissue by binding to GFR alpha3–RET, an artemin specific receptor in the MAP kinase pathway [39]. It has also been shown to attenuate neuropathic pain in individuals with spinal cord injuries and plays a protective role against deteriorating motor neurons in ALS patients [40,41]. To form the biocomposite, we then conjugated the laminin-artemin-bound templates with Type IV Collagen. It is well known that Type IV Collagen is a component of the basement membrane of vascular tissues in brain and forms mesh-like structures with advantageous mechanical properties. Furthermore, Type IV collagen promotes cell adhesion and stability [42]. 

Finally, emeraldine base polyaniline (PANI)—a conductive polymer—was incorporated to impart electric properties to the scaffold. PANI consists of repeat units of benzene rings which are separated by secondary amine groups and a quinoid ring system attached to imine groups [43]. The protonated form of emeraldine is conductive as it can form a semiquinone radical cation [44]. In a study where emeraldine polyaniline was blended with gelatin and then electrospun into nanofibers, the scaffold showed a marked increase in conductivity after incorporation of PANI [45]. Thus, we have created a new composite scaffold that consists of self-assembled nanoribbons, conjugated with key proteinaceous components to enhance growth and proliferation of neuronal cells as well as a conductive polymer, polyaniline to impart electrical properties. The formed scaffold demonstrated biodegradability, enhanced mechanical properties as well as promoted growth and proliferation of cortical cells and promoted axonal outgrowth Thus, these newly formed scaffolds may have potential applications in neural tissue engineering. 

## 2. Materials and Methods 

Amino acid isoleucine and 3,3-tetramethylene glutaric acid, dimethylformamide (DMF), *N*-Hydroxy Succinimide (NHS), 1-ethyl-3-(3-dimethylaminopropyl) carbodiimide (EDAC) and triethylamine (TEA), Bradford reagent, Bovine serum albumin (BSA) were purchased from Sigma Aldrich. Buffer solutions of various pH values were purchased from Fisher Scientific. Mouse laminin (sc-29012) and laminin alpha-2 antibody (B-4) were purchased from Santa Cruz Biotechnology. NHS-rhodamine was purchased from Thermo Scientific. Anti-collagen Type IV (rabbit) antibody was purchased from Rockland. Human artemin (category 4515-20, lot P70215) was purchased from Bio Vision. Type IV collagen (AG 19502) was purchased from Neuromics. Rat cortical cells (E18) and cell culture media (NbActiv4) were purchased from BrainBits. Neuroblast cell culture media and Glutamax were purchased from Gibco. Polyaniline was purchased from Ark Pharmaceuticals. Solvent *N*-methyl-2-Pyrrolidine was purchased from VWR. The digital multimeter model M-1000D was purchased from Elenco and a 1.62 mm diameter platinum electrode was purchased from Bioanalytical Systems Incorporated. 

### 2.1. Synthesis of Ile-TMG-Ile 

To TMG (1M) were added NHS (0.1M) and EDAC (0.1M) in DMF for activating the carboxylic acid groups of TMG. The mixture was stirred for one hour at 4 °C followed by the addition of Ile (2M). Two drops of TEA were added and the reaction mixture was stirred at 4 °C for 24 h. After 24 h, the solution was rotary evaporated to remove the solvent. The product obtained was found to be a white solid. The ESI-MS obtained by HPLC-MS (Agilent 6100 series, Santa Clara, CA, USA) showed a very weak M+ peak at m/z 411.2; peaks were also observed at m/z 952.4 and at m/z 805.5 due to the formation of oligmers. The strongest peak was seen at m/z 393.2 due to loss of hydroxyl radical. The product most likely undergoes McLafferty rearrangement, as expected for amides. Smaller fragments were observed at m/z = 360.2; 230.7, 361.2 and 115.0. Thus, in addition to Ile-TMG-Ile, side products that included oligomers were also formed. The product was recrystallized using methanol and dried under vacuum before further analysis. The yield of the product was found to 57.2%. The formation of the product was confirmed by ^1^H NMR spectroscopy using a Bruker 400 MHz NMR (Billerica, MA, USA) in deuterated DMSO with TMS as a solvent. ^1^H NMR (DMSO-d6) spectrum showed peaks at δ 0.9 (t, 6H); δ 1.2 (d, 6H); δ 1.4 (t, 2H); δ 1.7 (m, 4H); δ 2.3 (d, 2H); δ 2.7 (s, 4H); δ 2.9 (s, 4H); 3.4 (s, 4H); δ 8.1 (s, 2H); δ 12.2 (s, 2H). ^13^C NMR (DMSO-d6) showed peaks at δ 17.3; δ 22.2; 25.3; δ 29.6; δ 33.4; δ 39.5 δ 42.7; δ 45.9 and δ 174.2.

### 2.2. Self-Assembly of Ile-TMG-Ile Template

The synthesized product was allowed to self-assemble in buffer solutions of varying pH values. In general, the product (45 mM) was allowed to assemble under acidic (pH 4, potassium acid phthalate buffer); neutral (pH 7, potassium phosphate monobasic-sodium hydroxide buffer) and basic (pH 9, boric acid, potassium chloride, sodium hydroxide buffer) conditions over a period of three to four weeks at room temperature. The growth of assemblies was monitored by dynamic light scattering periodically. After four weeks of growth, the assemblies were centrifuged and washed thrice with deionized water to remove the buffer and left in deionized water for further analysis. 

### 2.3. Preparation of Scaffold

The washed Ile-TMG-Ile assemblies grown at pH 7 were utilized for preparation of scaffolds. An aqueous solution of the fibrillar assemblies (2 mM) was treated with EDAC (1 mM) and NHS (1 mM) for one hour at 4 °C to activate the free carboxylic groups in Ile-TMG-Ile. Mouse laminin (0.1 mg/ mL, 200 µL) was then added to the activated template. The mixture was stirred at 4 °C for 24 h to allow for adhesion of laminin. The laminin bound templates were then washed and centrifuged thrice with deionized water to remove any unbound laminin followed by addition of EDAC (1 mM) and NHS (1 mM) for one hour at 4 °C. To the laminin bound templates, artemin (0.1 mg/mL, 200 µL) was then added and shaken at 4 °C for 24 h and washed and centrifuged thrice to remove unbound artemin. The laminin and artemin functionalized construct was once again allowed to react with EDAC (1 mM) and NHS (1 mM) for one hour at 4 °C followed by the addition of Type IV collagen (0.1 mg/mL, 100 µL) and was shaken at 4 °C for 24 h. The biocomposite was washed and centrifuged to remove unbound collagen. The formed biocomposite scaffold was then vacuum dried. To the dried scaffold, polyaniline (PANI) (0.1 mg/mL) in *N*-methyl-2-Pyrrolidine (5 mL) was added and the mixture was shaken at 4 °C for 24 h and then centrifuged for three hours to remove unbound PANI. 

### 2.4. Binding Efficiency of Laminin, Artemin and Type IV Collagen on the Assemblies

The efficiency of binding of each of the protein components was examined by UV-Vis spectroscopy which was monitored at 272 nm and the absorbance before and after binding to each of the proteins was measured. The absorbance of laminin (0.1 mg/mL, 200 μL) was compared, with that of washed laminin bound assemblies which were prepared as described above. The volumes of the solutions were kept constant. Our results indicated 84.6% binding of laminin to the assemblies. Subsequently the protein concentration of laminin bound assemblies was determined using the Bradford method, based on a standard curve obtained for BSA (1 mg/mL). The concentration of laminin on the assemblies was found to be 2.5 μM. Similarly, upon binding with artemin, the binding efficiency was found to be 92.3% and the protein concentration was found to be 3.19 μM. Finally, for Type IV collagen, the binding efficiency was found to be 88.3% and the protein concentration after binding to Type IV Collagen was found to be 3.65 μM. 

### 2.5. In Vitro Biodegradability Studies

To examine the biodegradability of the formed scaffold, 45 mg of the scaffold was weighed in a petri dish to which 10 mL of simulated body fluid buffer (SBF) was added. The weight of the scaffold was measured every 10 h over a period of 22 days. In general, at each time point, the scaffold was rinsed with deionized water and air dried at room temperature before measurement of weight. The SBF was replaced as necessary each time with the same volume (10 mL). Studies were carried out in triplicate. The results were then analyzed as a function of time. The SBF was prepared according to previously established methods [46]. Briefly, to prepare the simulated body fluid (SBF) for biodegradability studies, 750 mL of distilled water was first brought to a constant temperature of 36.5 °C. The solution was constantly stirred while adding 7.996 g of NaCl, 0.350 g NaHCO_3_, 0.224 g KCl, 0.228 g K_2_HPO_4_ 3H_2_O, 0.305 g MgCl_2_ 6H_2_O, 40 mL HCl (1 M), 0.278 g CaCl_2_, 0.071 g Na_2_SO_4_ and 6.057 g (CH_2_OH)_3_CNH_2_. The pH was adjusted to 7.4 with dropwise addition of 1 M HCl. The total volume was then brought to 1 L using distilled water and then the solution was stored at 4 °C before use. 

### 2.6. Cell Studies

To examine cell viability, rat cortical cells (E18, lot, BrainBits) were cultured in NbActiv4 media (BrainBits) containing 1% 10,000 g/mL amphotericin and 100 units/mL penicillin and streptomycin. The cells were grown to confluence and kept in a humidified atmosphere of 5% CO_2_ at 37 °C. To examine the effects of the scaffold, cells were plated in 12-well Falcon polystyrene tissue culture plates at a density of 1 × 10^3^ cells per well. After allowing the cells to adhere to the well plates for two hours, scaffolds were added at varying concentration (6 μM, 10 μM and 13 μM). The scaffolds were allowed to interact with the cells for 24, 48 and 72 h. These studies were carried out in triplicate. After each allotted period of time, cell viability and growth was examined by via trypan blue method. Cortical cells in media alone were used as a control for this study. Once stained with trypan blue, live and dead cells were counted using a hemocytometer and averaged. The percent viability was then calculated as follows: (living cortical cells)/(living cortical cells + dead cortical cells) × 100. To observe the interactions of the cortical cells with the scaffolds, cells were plated and allowed to interact with scaffolds as before. The media was changed every 48 h. Images were taken using an AmScope IN200TA-P Inverted Tissue Culture Microscope (Irvine, CA, USA) with a USB camera at various magnifications every 24 h over a period of seven days.

### 2.7. Electrochemical Studies

We first examined the resistance of control PANI, PANI-bound scaffolds and the biocomposite scaffold in the absence of PANI and determined the conductivity. The resistance of the scaffolds was tested in HCl (1M). A digital multimeter, model M-1000D, from Elenco (Philadelphia, PA, USA) was then used to determine the resistance. To examine the electrochemical properties of the scaffold, we conducted cyclic voltammetry in the presence of PANI, PANI-bound scaffolds and the biocomposite scaffold before it was bound to PANI as control. To obtain I-V curves, the solutions were dried directly onto the 2.01 mm^2^ electrode using a vacuum pump for a period of 24 h, adding an addition layer every 6 h. A voltage cell was created using this working electrode, a platinum counter electrode, an Ag/AgCl reference electrode and 1M HCl. Prior to electrochemical measurements, nitrogen was bubbled into the cell for 20 min to remove dissolved oxygen from the solution. PowerSuite by Princeton Applied Research and a Princeton Applied Research potentiostat model 263A (Oakridge, TN, USA) were used to obtain I-V curves from a potential of −0.2 V to 0.9 V at 10 mV/s. 

### 2.8. Characterization 

#### 2.8.1. Fourier Transform Infrared (FTIR) Spectroscopy 

FTIR spectroscopy was conducted using a Thermo Scientific Nicolet IS50 FTIR (Waltham, MA, USA) with OMNIC software. In general, samples were run at a range of 400 cm^−1^ to 4000 cm^−1^ with 100 scans per sample and the data obtained was averaged. 

#### 2.8.2. Scanning Electron Microscopy (SEM) 

To examine the morphologies of the assemblies as well as the incorporation of each layer after conjugation, samples were air-dried on to carbon double stick tape and carbon coated to prevent charging. Samples were imaged at various magnifications between of 2 kV to 10 kV utilizing a Zeiss EVO MA10 scanning electron microscope (Thornwood, NY, USA). 

#### 2.8.3. Transmission Electron Microscopy (TEM)

In order to further elucidate the morphologies of the assemblies we also conducted TEM analysis using JEOL 1200 EX transmission electron microscope (Peabody, MA, USA). Samples were air-dried on to formvar/carbon 200 mesh copper grids overnight before analysis. Samples were imaged at various magnifications at 80 KeV. 

#### 2.8.4. Dynamic Light Scattering (DLS) 

To monitor the growth of the assemblies, we conducted DLS using a NICOMP 380 ZLS sizer (Willow Grove, PA, USA). Samples were diluted to appropriate concentrations and each sample was run at least three times and the data obtained was averaged. 

#### 2.8.5. Atomic Force Microscopy (AFM)

We examined the nanoscale morphology of the assemblies after conjugation with each layer using a Bruker Multimode 8 AFM (Santa Barbara, CA, USA). Furthermore, the mechanical properties of the scaffolds were also determined by conducting Peak Force Microscopy. The tip was moved to various points (at least five points per sample) on each sample and the values obtained were averaged. Young’s Modulus was determined by fitting the data into a Hertzian Model. In general, we used RTESPA-175 Antimony (n) doped Si tip with a spring constant of 40 N/m. 

#### 2.8.6. Differential Scanning Calorimetry (DSC)

To explore the thermal phase changes of the scaffolds, we conducted DSC analysis. For each analysis, 0.1 mg samples were dried under vacuum and weighed. We examined the phase changes for each layer of the scaffold using a TA instruments, model Q200 instrument (New Castle, DE, USA) at a temperature range of 0 °C to 250 °C at scanning rate of 10 °C per minute. 

#### 2.8.7. Fluorescence Microscopy 

Fluorescence microscopy was carried out to examine the interactions of FITC labeled laminin -2 antibody (B-4) with laminin bound assemblies and rhodamine labeled Type IV collagen with the biocomposites using a Phase Contrast Amscope Fluorescence Inverted Microscope (Irvine, CA, USA). To prepare samples for binding with laminin antibody, the laminin bound assemblies were washed and centrifuged with deionized water followed by the addition of BSA Blocker solution (1% BSA) in tris buffered saline to prevent non-specific binding. The sample was vortexed for two minutes and allowed to incubate at room temperature for 4 h. The sample was then centrifuged and washed once with TBS followed by washing with deionized water. To the sample, we then added FITC labeled laminin -2 antibody (B-4) (50 μg/mL). The sample was incubated overnight at 4 °C. Samples were then washed and centrifuged with deionized water and imaged on poly-L-lysine coated glass slides which was covered by a coverslip. Samples were then excited at 450 nm. A similar protocol was followed for examining the interactions of rhodamine labeled collagen IV antibody, where in the collagen IV-artemin-laminin-bound Ile-TMG-Ile assemblies were first washed and centrifuged, followed by the addition of 1% BSA blocking agent before incubation with the antibody. Finally samples were transferred to glass-slides covered with coverslips and imaged at 588 nm excitation. 

#### 2.8.8. UV-Vis Spectroscopy

To determine the binding efficiency and protein concentrations of the biocomposite assemblies after each layer of protein (laminin, artemin and Type IV collagen) was added, we carried out UV-Vis spectroscopy using a Nanodrop 2000 spectrophotometer (Waltham, MA, USA). 

### 2.9. Statistical Analysis

We used two-tailed Student’s t-test for carrying out statistical analysis. Studies were carried out in triplicate (n = 3). Data are presented as the mean value ± standard deviation (SD) of each sample group. 

## 3. Results and Discussion 

### 3.1. Self-Assembly of Ile-TMG-Ile 

Molecular self-assembly of biomolecules transpires through weak, non-covalent interactions that include electrostatic, hydrophobic interactions, hydrogen bonds, van der Waals interactions and π–π stacking forces that result in the formation of stable and functional supramolecular structures [47]. Self-assembling peptides, in particular form unique supramolecular assemblies and offer several advantages. Thus, such materials pose a plethora of applications in tissue engineering as they are highly biocompatible and modifiable [48]. 

**Figure 1:**
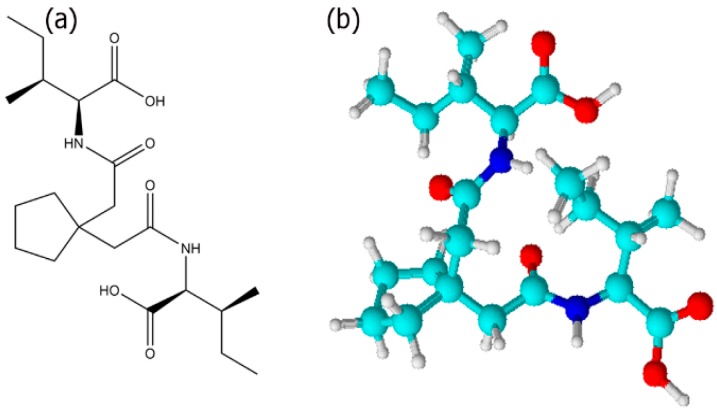
(**a**) Chemical structure of Ile-TMG-Ile; (**b**) ball and stick model.

In this work, we designed a new bolaamphiphile by conjugating the amino acid Ile with the dicarboxylic acid 3,3 tetramethylene glutaric acid (TMG) resulting the in the formation of (2S,2'S,3S,3'S)-2,2'-((2,2'-(cyclopentane-1,1-diyl)bis(acetyl))bis(azanediyl))bis(3-methylpentanoic cid), abbreviated as Ile-TMG-Ile (Figure 1). In previous work, peptide based bolaamphiphiles containing amino acid moieties such as glycine, conjugated with dicarboxylic acids such as azelaic acid have been shown to self-assemble into nano and microtubes with closed ends or single layered sheets [49]. In an earlier study, we have shown that when phenylalanine was conjugated with dicarboxylic acids of different chain lengths, supramolecular assemblies of a variety of morphologies were formed depending upon the growth conditions used [50]. It has also been shown that peptide amphiphiles containing N-terminus palmitoylated groups such as CH_3_-(CH_2_)_14_CO-NH-X-Ala_3_-Glu_4_-CO-NH_2_, where in the amino acid X was varied between Ile, Phe or Val, self-assembled into micelles, nanoribbons or nanofibers depending upon the pH of the growth conditions [51]. 

**Figure 2:**
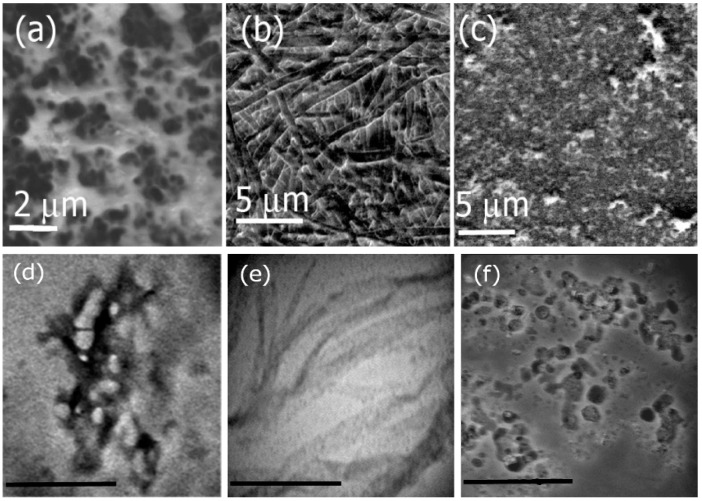
SEM images of Ile-TMG-Ile assemblies formed at (**a**) pH 4; (**b**) pH 7 and at (**c**) pH 9. TEM images of the assemblies are shown at (**d**) pH 4 (scale bar = 500 nm); (**e**) pH 7 (scale bar = 2 μm) and (**f**) pH 9 (scale bar = 1 μm nm).

Herein, we examined the self-assembly of Ile-TMG-Ile at pH values of 4 through 9 for a period of four weeks. To examine the morphologies of the formed assemblies, we conducted SEM and TEM microscopy. Figure 2 shows the SEM and TEM images of assemblies formed at varying pH. SEM analysis at pH 4 (Figure 2a) indicates the formation of short, thick nanofibers in the diameter range of 500 nm to 1 μm, while at pH 7 (Figure 2b) we observed the formation of long, multilayered nanoribbons several micrometers in length, with an average diameter of 500 nm to 1 μm. Figure 2c shows the structures of the assemblies formed under basic conditions (pH 9). Results indicated that under basic conditions, structures of a variety of shapes and sizes (spherical micelles, microtubes and fibers) in the range of 2 μm to 5 μm in diameter were formed. Corresponding TEM images, also indicated similar morphologies as shown in Figure 2d–f which correspond to assemblies formed at pH 4, 7 and 9 respectively. The sizes obtained by the TEM analysis, indicate that the average diameter of the nanofibers formed at pH 4 was found to be 20 nm, while those grown at pH 7 were in the size range of 500 nm to 1μm. The assemblies formed at pH 9 were found to be in the range of 200 nm to 500 nm. The size differences between TEM and SEM are attributed to the fact that the assemblies are intrinsically multiscale in nature and, vary in sizes. The TEM images display higher resolution and smaller sample sizes are most likely revealed by TEM analysis. Overall, self-assembly of Ile-TMG-Ile was found to be pH dependent. 

The formation of short, thick nanofibers under acidic conditions is attributed to higher H-bonding interactions under acidic conditions as the carboxylic groups of the side chain isoleucines are likely to be protonated under those conditions. Additionally, assembly formation is promoted due to intermolecular H-bonding interactions between the –NH and O=C groups of the amide groups of the bolaamphiphile. Studies conducted previously with peptide amphiphiles such as bis(*N*-α-amido-glycylglycine)-1,7-heptane dicarboxylate have shown that at a pH range of 4 to 5, the formation of nanotubes is promoted due to higher H-bonding interactions and beta-sheet formation [52]. In the case of Ile-TMG-Ile, it is likely that beta-sheet formation is promoted, particularly due to the hydrophobicity of the Ile moieties which have been known to induce nanofiber formation [53]. Under neutral conditions, there appears to be a transition between beta-sheet structures to random coil due to changes in H-bonding interactions as the carboxyl groups are progressively deprotonated, resulting in the formation of uniform nanoribbons. Similar phenomena have been observed in the case of bola-glycolipids where changes in morphologies of supramolecular structures were observed, resulting in nanoribbon formation under neutral conditions. This was primarily attributed to a combination of hydrophobic interactions, chirality as well as well as changes in pH [54]. Under basic conditions, we observed a mixture of nanostructures as under those conditions the Ile-TMG-Ile bolaamphiphle is completely deprotonated and H-bonding is significantly diminished. Although C=O---NH amide H-bonding still exists under basic conditions, the carboxylate groups are negatively charged under those conditions and may result in repulsion between the negatively charged carboxylate groups. Thus, a variety of structures including micelles and few fibrillar structures are formed due to a combination of hydrophobic interactions, as well as amide-amide H-bonding and uniform assemblies are not formed. 

**Figure 3:**
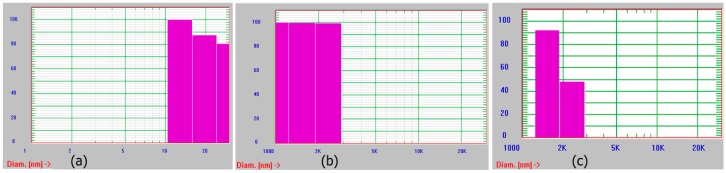
Dynamic light scattering analyses of assemblies formed at (**a**) pH 4; (**b**) pH 7 and (**c**) pH 9.

We also monitored the growth of assemblies periodically using dynamic light scattering in all cases and over time. Results obtained after two weeks of growth are shown in Figure 3. As seen in the figure, the assemblies obtained were polydisperse. This is most likely because the assemblies are not uniform. They are mostly fibrillar, or ribbon shaped (in the case of assemblies grown at pH 4 and pH 7) or display a variety of morphologies as seen in the case of assemblies grown at pH 9. It is likely that aggregates of the assemblies at different stages of growth are observed. Overall, due to the formation of ribbon like structures under neutral conditions, we selected those assemblies for preparation of the scaffold. 

### 3.2. Functionalization of Ile-TMG-Ile and Preparation of Scaffold

**Figure 4:**
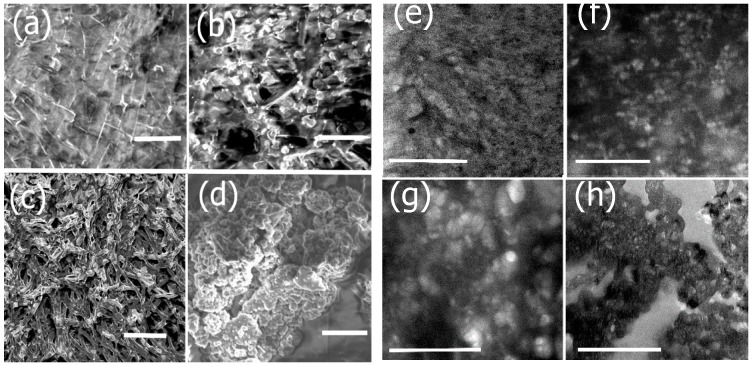
SEM and TEM images showing morphology changes after functionalization with each component. (**a**) SEM image of nanoribbons functionalized with laminin (scale bar = 2 μm); (**b**) SEM image showing subsequent conjugation with Artemin (scale bar = 2 μm); (**c**) SEM image after incorporation of Type IV collagen to laminin-artemin-bound nanoribbons (scale bar = 5 μm); (**d**) SEM image after incorporation of PANI to the functionalized biocomposite (scale bar = 5 μm). (**e**) TEM image of nanoribbons functionalized with laminin (scale bar = 2 μm); (**f**) TEM image showing subsequent conjugation with Artemin (scale bar = 2 μm); (**g**) TEM image after incorporation of Type IV collagen to laminin-artemin-bound nanoribbons (scale bar = 3 μm); (**h**) TEM image after incorporation of PANI to the functionalized biocomposite (scale bar = 3 μm).

To prepare the scaffolds that can be tailored for potential neural TE applications, we incorporated protein constituents that may aid in neural tissue regeneration due to their specific functional properties. The conjugation of each component was examined by SEM and TEM imaging as shown in Figure 4. We first conjugated the washed nanoribbons with laminin, a major component of the ECM of neural tissue. Upon incorporation of laminin, changes in morphology were observed (Figure 4a). The SEM image of laminin bound assemblies showed a relatively rough, gelatinous coating on the nanoribbons compared to the smooth surfaces in the absence of laminin as seen in Figure 2b. The corresponding TEM image (Figure 4e) showed a fibrous mesh like network upon conjugation with laminin. In general, laminin consists of both globular and rod-shaped domains and forms alpha-helical coiled coil structures [55]. Upon conjugation with the nanoribbons, laminin binds to nanoribbons, forming a gelatinous network intertwined with the nanoribbons. In general, laminin has been known to polymerize and form laminin networks, in cells due to interactions between the α-short arms of the amino terminal domain and β and γ-short arms of laminin [56]. It is likely that it wraps around Ile-TMG-Ile nanoribbon assemblies upon conjugation resulting in the mesh like networks. 

Upon conjugation with artemin, further morphology changes were observed. In the SEM image (Figure 4b), we observed the incorporation of globular, rosette like structures throughout the gelatinous matrix. Similar structures were observed in the in the corresponding TEM image (Figure 4f). Previous studies have revealed that artemin monomers tend to self-assemble into rosette-like oligomers [57] and it is also known to be an exceptionally stable neurotrophic factor that can induce changes in the folding process of proteins as it functions as a molecular chaperone [58]. Thus, the morphology changes observed on the surfaces of the laminin bound nanoribbons further confirm the successful conjugation of artemin. We then conjugated the composite nanoribbons with Type IV collagen (Figure 4c,g). SEM and TEM images confirmed morphological changes after incorporation of Type IV collagen as the formation of large fibrillar mesh like structures were observed, integrated with rosette structures of artemin. In several studies, it has been shown that collagens tend to form long fibrillar structures, due to the formation of triple-helices and impart structural integrity to scaffolds [59]. Thus, our results confirm the formation of the biocomposite nanoribbons integrated with laminin, artemin and Type IV collagen. To impart electrical properties, essential for developing scaffolds for neural TE, we then incubated the conductive polymer polyaniline (PANI) with the biocomposite scaffolds. Previous studies have shown that PANI in the presence of dopants can self-assemble into nanostructures [60]. The presence of the amine groups of PANI, allow for electrostatic and H-bonding interactions between the carbonyl groups of the protein bound nanoribbons and PANI. Additionally, stacking interactions with the aromatic ring systems of PANI are also promoted between the proline and hydroxyproline moieties of Type IV Collagen. Distinct changes in morphology were observed upon incorporation of PANI into the biocomposite (Figure 4d,h) showing the formation of aggregates of PANI on the scaffold indicating its successful assimilation.

### 3.3. FTIR Spectroscopy

**Figure 5:**
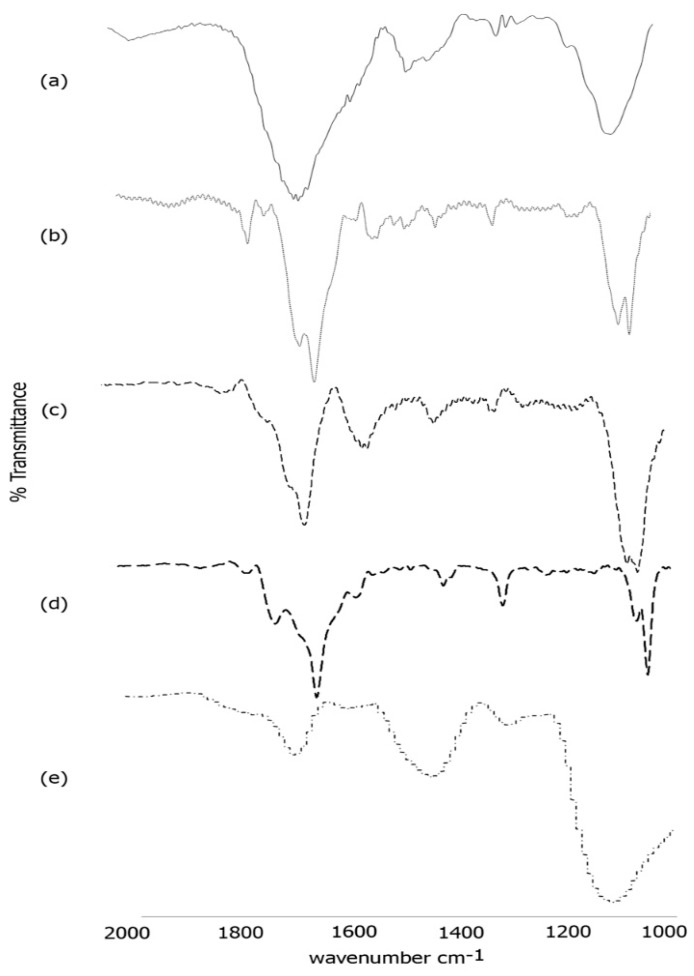
FTIR spectra of (**a**) self-assembled Ile-TMG-Ile nanoribbons; (**b**) Nanoribbons bound to laminin; (**c**) Artemin conjugated with laminin bound nanoribbons; (**d**) Type IV collagen conjugated with artemin-laminin bound nanoribbons; (**e**) PANI bound to Type IV collagen-artemin-laminin bound nanoribbons.

To further confirm the formation of the scaffold, we conducted FTIR spectroscopy (Figure 5). As shown in Figure 5a, the self-assembled template nanoribbons showed characteristic peaks in the amide I region at 1658 cm^−1^ and at 1650 cm^−1^ with a shoulder at 1634 cm^−1^ along with peaks at 1450 cm^−1^ and 1413 cm^−1^ in the amide II region. These peaks are indicative of formation of a mix of random coil, alpha helical and beta-sheet structures [61] that resulted in the formation of nanoribbons. Previous studies using protein analogs with C terminus isoleucine are consistent with these findings of mostly alpha helical and random coil structures [62]. Additionally, a strong peak was observed at 1286 cm^−1^ and at 1075 cm^−1^, attributed to C-O and C-H stretching respectively. Upon conjugation with laminin (Figure 5b), the amide I peaks were observed at 1656 cm^−1^ and at 1628 cm^−1^ indicating increased beta-sheet formation along with the presence of alpha helices. The amide II peaks were observed at 1550 cm^−1^ and at 1519 cm^−1^ while the C-O and C-H stretching peaks were seen at 1295 cm^−1^ and at 1059 cm^−1^ respectively [63]. It has been reported that laminins generally tend to polymerize into sheet like structures [64], which is consistent with our results. Furthermore, similar shifts were also seen after incorporation of laminin onto a poly(l-lactide-co- glycolide) scaffold, indicating successful conjugation of laminin with the nanoribbons [65]. After conjugation with artemin (Figure 5c), further shifts were observed. The amide I band was shifted to 1632 cm^−1^ with a shoulder at 1662 cm^−1^ while the amide II peaks shifted to 1535 cm^−1^ and 1514 cm^−1^. The C-O and C-H stretching peaks were observed at 1299 cm^−1^ and at 1064 cm^−1^ with a shoulder at 1054 cm^−1^ respectively. These peaks are indicative of increase in beta-sheets along with the appearance of beta-turn structure.

Upon incorporation of Type IV collagen (Figure 5d), the FTIR spectra showed peaks in the amide I region at 1629 cm^−1^ with a shoulder at 1652 cm^−1^ and a peak at 1699 cm^−1^. The amide II peak was found to be at 1558 cm^−1^ while the C-O and C-H stretching peaks were observed at 1293 cm^−1^ and at 1035 cm^−1^ respectively. These changes confirm the incorporation of Type IV collagen. Furthermore, the secondary structure reveals the presence of alpha-helical content along with beta-strands and β and γ-turns [66] due to blending of Type IV collagen with artemin-laminin bound nanoribbons. Distinct changes were observed upon incorporation of PANI. As seen in Figure 4e, peaks were found at 1680 cm^−1^, 1560 cm^−1^ and at 1430cm^−1^, 1280 cm^−1^ and at 1112 cm^−1^. The peaks at 1630 cm^−1^ at 1450 cm^−1^ are indicative of vibrations from quinoid rings and benzenoid ring systems as seen in PANI bound polystyrene nanocomposites [67]. These results further confirm the formation of the composite scaffold.

### 3.4. Fluorescence Microscopy

**Figure 6:**
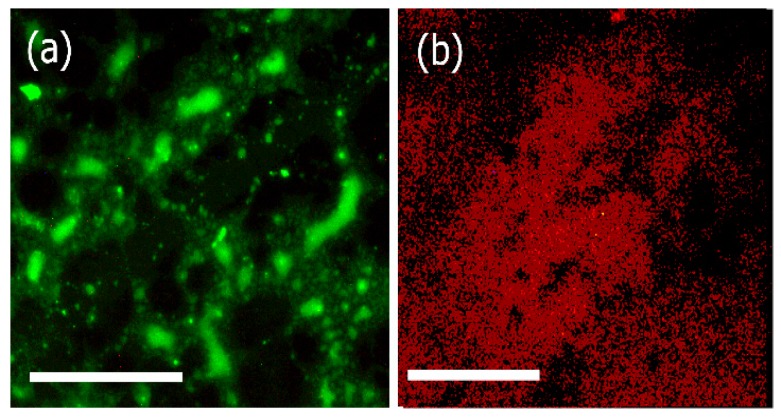
Fluorescence microscopy images of (**a**) FITC labeled laminin α-2 antibody bound to laminin bound assemblies; (**b**) Rhodamine labeled collagen IV antibody bound to biocomposite assemblies (collagen IV-artemin-laminin bound Ile-TMG-Ile). Scale bars = 20 μm.

In order to confirm that the proteins retained biological activity after conjugation with the assemblies, we examined the binding affinity of the laminin bound assemblies as well as the Type IV collagen bound biocomposites with corresponding antibodies. We used FITC labeled laminin -2 antibody (B-4) for laminin bound assemblies and rhodamine labeled anti-collagen (Type IV) antibody for Type IV collagen bound biocomposites respectively and examined the interactions using fluorescence microscopy. In previous work, it has been shown that conjugation of different biological moieties including specific peptide sequences such as RGD, TAT peptides, or proteins such as transferrin, antibodies with nanomaterials not only increases the stability of the proteins but also enhances the applications of the nanomaterials themselves [68]. Furthermore, such conjugations allow for interactions with cell receptors or biological membranes, thereby promoting the use of such materials for a variety of biomedical applications. As shown in Figure 6, FITC conjugated anti-laminin bound antibodies efficiently bound to the laminin bound assemblies, (Figure 6a) and rhodamine conjugated anti-collagen IV antibodies bound to the biocomposite (Figure 6b). These results confirmed that laminin and Collagen IV retained their biological activity upon binding with the assemblies. 

### 3.5. Differential Scanning Calorimetry

**Figure 7:**
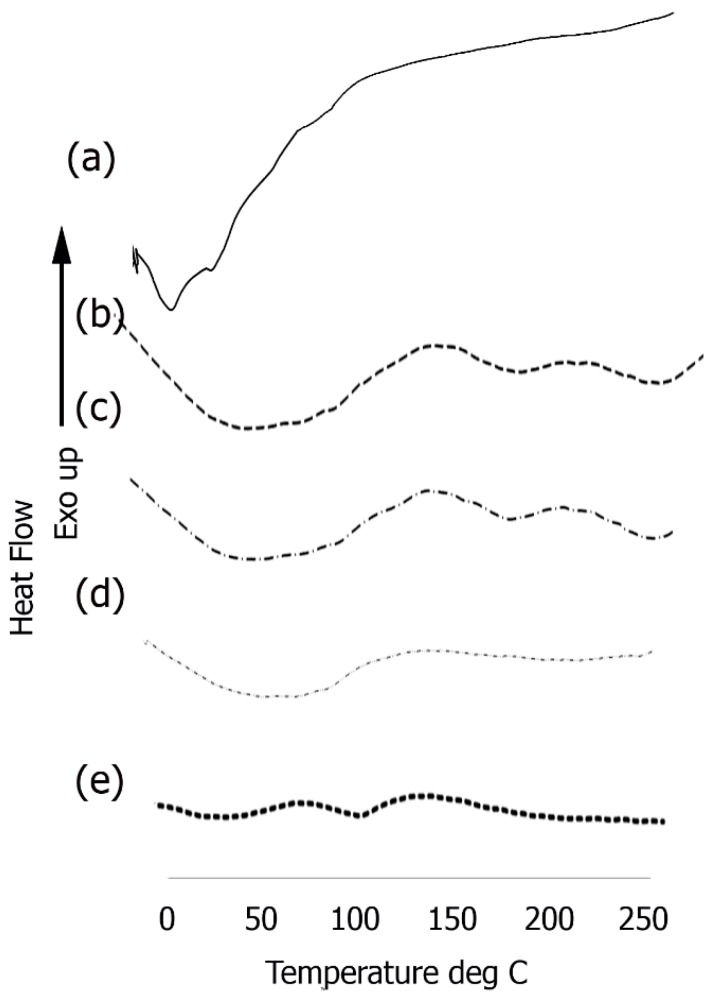
DSC thermograms of (**a**) self-assembled Ile-TMG-Ile nanoribbons; (**b**) Nanoribbons bound to laminin; (**c**) Artemin conjugated with laminin bound nanoribbons; (**d**) Type IV collagen conjugated with artemin-laminin bound nanoribbons; (**e**) PANI bound to Type IV collagen-artemin-laminin bound nanoribbons.

In order to explore the thermal properties, we examined phase changes of the assemblies before and after conjugation with each component utilized in the formation of the scaffold (Figure 7). Short endothermic peaks were observed in the case of the Ile-TMG-Ile assemblies (Figure 7a) at 14.9 °C and at 36.7 °C followed by another endothermic peak at 99.6 °C, due to loss of loosely bound water. After functionalization with laminin, (Figure 7b) a large, broad endothermic peak was observed in the temperature range of 50 °C to 100 °C, followed by shallow endothermic peaks at 172.4 °C and at 232.1 °C. The significantly broad endothermic peak at the lower temperature is indicative loss of free water, due to the presence of hydrophilic amino acids in the protein. This is primarily due to the fact that hydrophilic groups can cause significant hydration and upon heating, changes in H-bonding interactions and consequently conformation changes in the laminin bound nanoribbons occur. This is a common occurrence in self-assembled peptides and proteins due to the rearrangement of components because of changes in inter and intra-molecular interactions [69]. The short peak at 171.4 °C is likely due to thermal melting, followed by crystallization at a higher temperature (232.1 °C). After further functionalization with artemin (Figure 7c) a similar broad endothermic peak was observed in the temperature range of 50 °C to 100 °C, (though the intensity was lower). Slight shifts were observed in the higher temperature range, with the thermal melting appearing at 172.6 °C and the subsequent short crystallization peak was observed at 242.1 °C. Upon binding with Type IV collagen, (Figure 7d) the composite once again showed a broad endothermic peak, between 50 °C to 100 °C, though the intensity of the peak was lesser than the previous layer, due to higher cross-linking in the presence of collagen and notably, peaks at higher temperature were diminished. 

However, a significant change was observed upon incorporation of PANI (Figure 7e), where in the intensity of the broad endothermic peaks seen in the protein bound scaffold was significantly reduced. Relatively short endothermic peaks are observed at 52 °C and at 102 °C due loss of loosely bound water and no other significant peaks are observed. PANI is significantly hydrophobic compared to the proteinaceous components, resulting in shallow peaks due to loss of free unbound water. These results further confirm the integration of PANI. 

### 3.6. Mechanical and Surface Properties

It is paramount that the designed scaffolds should be able to bear force loads in order to adequately support seeded cells and potentially boost the formation of new tissue. We utilized peak force microscopy to determine the mechanical properties of the scaffold. In general, nanoindentation was carried out using AFM to examine the changes in attractive and repulsive forces and the depth of indentation as the tip of the cantilever contacts the sample and is deformed. At least three to five points were selected for each sample to obtain force vs. separation curves and the data obtained was fit into Hertzian model to obtain the Young’s modulus. The results obtained for average Young’s Modulus values after incorporation of each of the components of the scaffold are shown in Table 1. 

**Figure 8:**
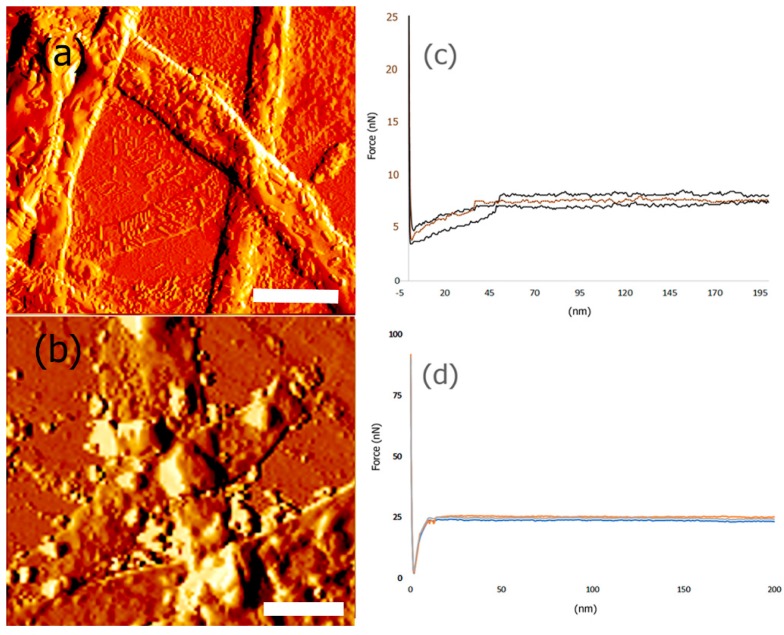
AFM amplitude image of (**a**) biocomposite nanoribbons bound to laminin, artemin and collagen. Scale Bar = 10 μm; (**b**) nanoribbons bound to laminin, artemin and collagen and PANI. Scale Bar = 5 μm. (**c**) Force curves obtained for biocomposite nanoribbons bound to laminin, artemin and collagen; (**d**) Force curves obtained for nanoribbons bound to laminin, artemin and collagen and PANI.

These results indicate that as each protein layer was conjugated with the nanoribbons, the Young’s Modulus (YM) was found to increase, demonstrating that each protein component consecutively increased the stiffness and mechanical strength of the scaffold. The YM values obtained were for dried scaffolds alone and are most likely higher than those one would expect for wet scaffolds, under in vivo or in vitro conditions. Previous nanoindentation studies have shown that native single collagen fibrils have an YM value in the range of 1 to 2 GPa [70]. It has also been reported that protein structures and self-assembled collagen based constructs designed to have properties to mimic the extracellular matrix display Young’s Moduli averaging 1.2 GPa [71]. We also determined the Young’s Modulus of control Type IV collagen by nanoindentation and the YM was found to be 532.2 ± 3 MPa, while control PANI films were found to have a YM of 1.52 ± 5 GPa. Thus, our results indicate that for the protein functionalized nanoribbon biocomposite, the overall mechanical strength of the scaffold increases due to the multi-component nature of the scaffold. Furthermore, it was found that the highest YM value was obtained after incorporation of PANI. A comparison of AFM images of scaffolds before and after incorporation of PANI and the corresponding force curves are shown in Figure 8. 

AFM topography images indicate that the surface of the bicomposite before binding to PANI (Figure 8a) shows a more fibrillar structure, due to top layer of the scaffold being Type IV collagen, while after binding to PANI, more globular structures were observed to be deposited on the scaffold (Figure 8b). These results indicate changes in surface roughness and morphology of the scaffold after incorporation of PANI. Additionally, the force-curves also showed significant changes upon binding with PANI (Figure 8c,d). These results are consistent with previous nanoindentation studies, where it has been demonstrated that when PANI was deposited on vertical arrays of carbon nanotubes (CNTs), the Young’s Modulus value dramatically increased due to strong electrostatic and π–π stacking interactions with CNTs [72]. It is expected that similar interactions occur between the biocomposite and PANI that leads to a higher YM. In general, the Young’s Modulus values of pyrrolidinone and polyaniline films have been reported to be in the range of 200 MPa–5 GPa [73]. Thus, our results after incorporation of PANI with the biocomposite scaffold are within the values reported previously in the literature. 

We also probed the changes in the surface roughness of the scaffold before and after incorporation of PANI. In general, it is known that surface roughness plays a key role in cell adhesion of scaffolds [74] For instance, it has been shown that silk fibroin bound PLA fibers with higher surface roughness promoted increased growth and adhesion of osteoblast cells [75]. For the biocomposite scaffold before incorporation of PANI, the average surface roughness (Ra) was found to be 154 nm, while the maximum roughness (Rmax) was determined to be 209 nm. We found that incorporation of PANI resulted in a significant increase in the surface roughness. The Ra was found to be 377 nm and the Rmax was found to be 2065 nm, further confirming the formation of the composite. 

### 3.7. In Vitro Biodegradability Studies

**Figure 9:**
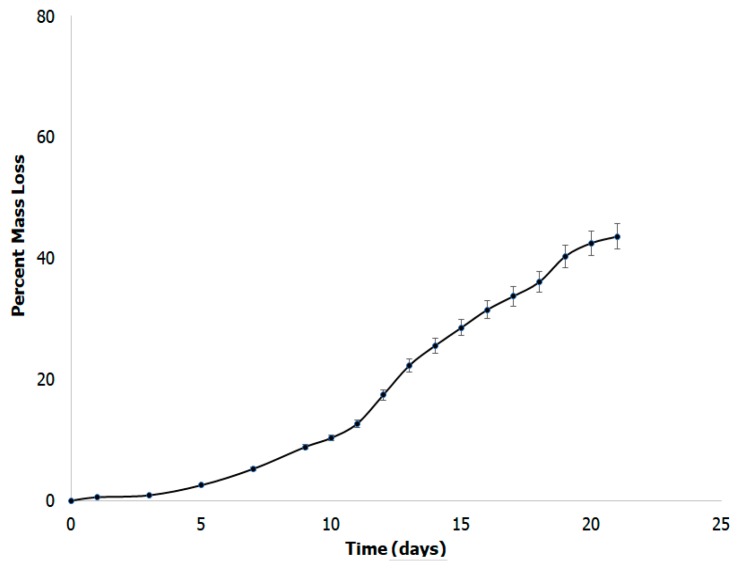
Biodegradation studies of the scaffolds showing mass loss over a period of 20 days.

When developing a scaffold for tissue engineering, the biodegradability of a scaffold plays a vital role. It has been reported that biodegradability promotes growth and proliferation of cells, as well as production of native ECM [76]. In comparison to non-biodegradable scaffolds, biodegradable composites are able to aid in the growth of new tissues at a highly expedited rate due to the increased proliferation and lack of hindrance for cell growth [77]. We examined the biodegradability of the formed scaffold in simulated body fluid buffer in order to mimic in vivo conditions. Figure 9 shows the results obtained over a period of three weeks in simulated body fluid. The scaffold shows a mass loss of 48.7% after 22 days. Overall, the scaffold showed degradation at a moderate rate, which is preferential to highly vascular nervous tissue [78]. This result is consistent with other polymer and basement membrane mimicking scaffolds for tissue engineering; such as, poly(3-hydroxybutyric acid) with chitin and chitosan which demonstrated biodegradability from 45–70% and collagen, hyaluronic acid and gelatin scaffolds which demonstrated 45% biodegradability [79]. 

### 3.8. Cell Studies

**Figure 10:**
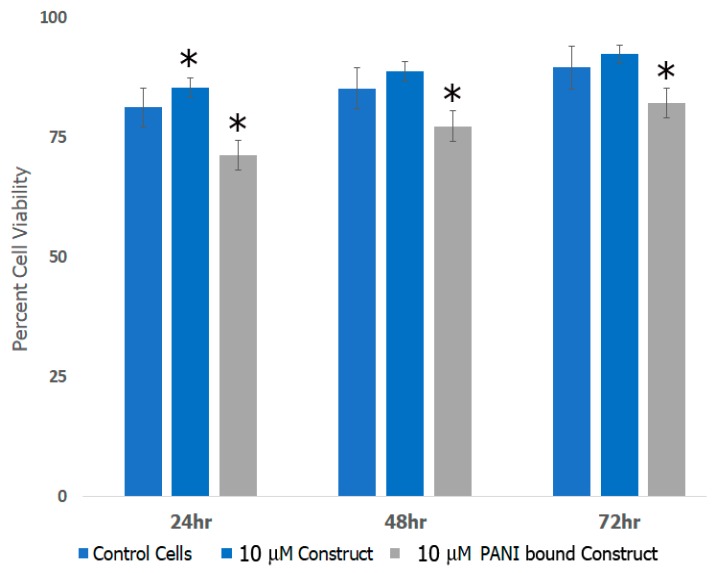
Cortical cell viability in the presence of scaffold constructs before and after incorporation of PANI. (* = p < 0.05 was determined to be statistically significant).

To examine if the formed scaffolds would be suitable for applications in neural TE, we conducted cell viability studies with rat neural cortical cells. We examined the effects of scaffolds before and after incorporation of PANI as shown in Figure 10. The data shown are representative of results obtained in the presence of 10 μM scaffolds in comparison with control untreated cells. Our results indicate that the cells continued to proliferate over a period of 72 h in the presence of scaffolds. Although cell proliferation continued over time, the rate of cell growth for the PANI bound construct was lower compared to the controls due to the known cytotoxicity of PANI [80]. This is most likely that proliferation continued due to the synergistic effects of the protein components of the scaffolds, which play a role in enhancing biocompatibility of the PANI bound scaffold. Previous studies have shown that laminin bound nanofibers significantly increased the attachment and neurite extension of cells in vitro [81] while artemin promotes axonal growth in damaged neural tissue, as well as regeneration of sensory neurons [82]. Other research has shown that after damage, artemin increases survivability of injured neurons [83]. The incorporation of Type IV collagen increases the influx of nutrients to growing and damaged neural tissue, increasing new growth and proliferation. We also studied the growth and proliferation in the presence of 6 μM and 13 μM scaffolds which showed similar trends of cell viability (data not shown). In general, no significant differences were observed in the proliferation at varying concentrations of the scaffold. These results indicated growth and proliferation of cortical cells continued and were comparative to the control throughout various time periods and concentrations of the construct.

**Figure 11:**
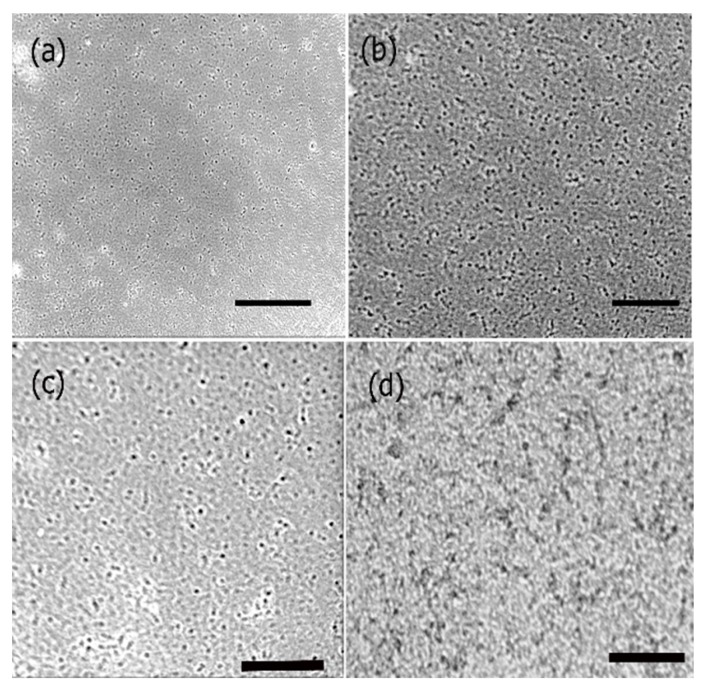
Phase contrast microscopy images showing the growth of neural cortical cells (**a**) control cells after 24 h; (**b**) control cells after 72 h; (**c**) cells with PANI bound scaffolds after 48 h and (**d**) cells with PANI bound scaffolds after 7 days. Scale bars: (**a**) 50 μm; (**b**) 50 μm; (**c**) 30 μm; (**d**) 50 μm.

To further investigate the cell proliferation of neural cortical cells and morphologies in the presence and absence of PANI bound scaffolds, we conducted phase contrast optical microscopy studies. The results obtained are shown in Figure 11. As seen in the figure, there was a major difference in the morphologies of the cells grown in the presence of PANI bound scaffolds over a period of seven days in comparison to those after 48 h. As shown in Figure 11a,b, in the absence of scaffold, cells continued to proliferate over time. However, we did not observe cluster formation or axonal outgrowths. Upon incubation with PANI bound scaffolds. After 48 h (Figure 11c), the cells appeared to relatively more elongated compared to controls However after seven days, we observed cluster formation and axonal outgrowth (Figure 11d). These results indicated that the cells efficiently continued to proliferate in the presence of the PANI bound scaffolds and enhanced neural cell growth as well as cell-cell adhesion, further confirming that the scaffolds were conducive to forming cell-scaffold matrices and provide an environment for the growth and support of neural cortical cells. 

### 3.9. Cyclic Voltammetry

**Figure 12:**
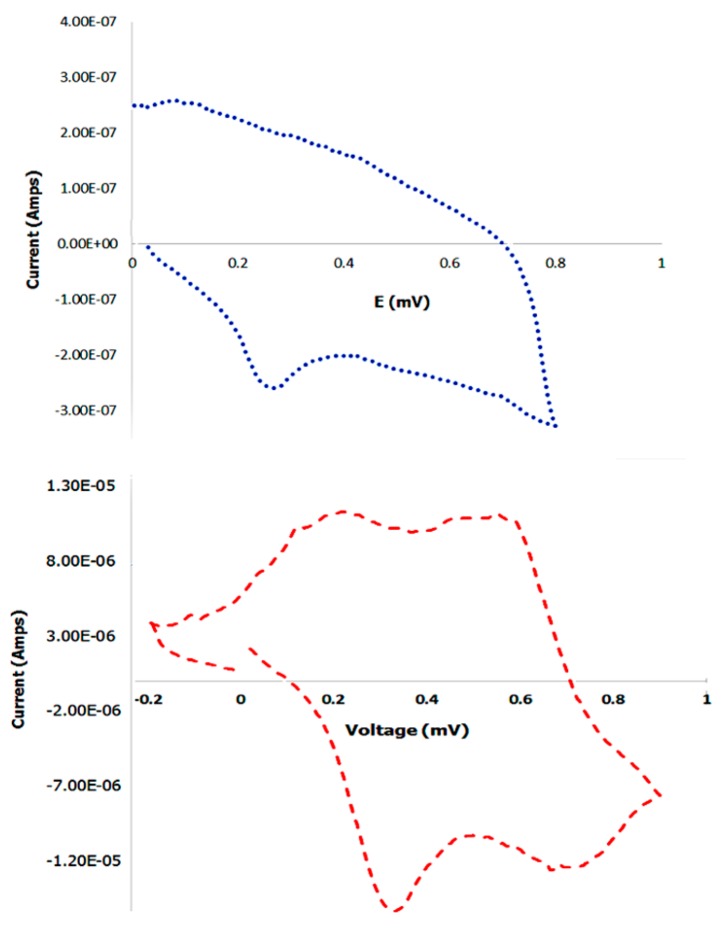
Comparison of cyclic voltagrams of control PANI (top) and PANI bound scaffold (bottom).

To examine the electrochemical properties of the PANI bound scaffold, cyclic voltammetry was conducted. Prior to performing the experiment, the control PANI or the scaffold bound PANI were dried onto the platinum electrodes in vacuum overnight. After connecting the electrodes to the potentiostat, nitrogen was bubbled into the HCl cell solution to remove oxygen from the solution. Potential between −0.2 V to 0.9 V was applied at 10 mV/s to obtain I-V curves. At a voltage of 0.1 mV, an anodic oxidation peak was observed of approximately 2.6 × 10^−7^ Amps current, with a corresponding cathodic reduction peak at 0.27 mV of approximately −2.85 × 10^−7^ Amps current in the case of the control PANI. Cyclic voltammetry was then conducted with scaffold bound PANI. Our results indicated that oxidation peaks were observed at 0.213 mV at 1.13 × 10^−5^ amps current and at 0.56 mV at 1.12 × 10^−5^ Amps which correspond to the lecuoemeraldine-emeraldine and emeraldine-pernigraniline oxidation processes [84]. The reduction peaks were observed at cathodic currents of −1.39 × 10^−5^ Amps and at −1.54 × 10^−5^ Amps at 0.67 mV and at 0.32 mV respectively. These results are shown in Figure 12. Similar cyclic voltagrams have been observed for electrospun polycaprolactone and polyaniline fibers used in skeletal tissue engineering [85].

Cyclic voltammetry of polyaniline-carbon nanotubes also yielded similar voltammograms [86]. We also compared the cyclic voltagrams with scaffolds formed before incorporation of PANI which did not show any peaks (data not shown). To further examine the electrical properties of the scaffolds, we measured the resistance of the scaffolds to determine the conductivity. Our results showed that the PANI bound scaffolds displayed a conductance of 1.5 × 10^−3^ S/cm; while the scaffolds in the absence of PANI displayed zero conductivity. Control PANI displayed a conductivity of 2.0 × 10^−3^ S/cm, which was higher than the PANI bound scaffolds. Overall, these results are indicative that upon binding to PANI the scaffolds display electrical properties, compared to absence of PANI, which is essential for scaffolds for neural tissue regeneration. 

## 4. Conclusions

In this work, 3,3 tetramethyleneglutaric acid and isoleucine were coupled and allowed to self-assemble into a nanoribbon matrix. The nanoribbons were then functionalized with laminin, a primary component in the neural cell basement membrane, along with artemin a glial cell line derived neurotropic factor and Type IV collagen another key component of the ECM of neural tissues. To impart electrical properties, we then incorporated polyaniline a conductive polymer into the scaffold matrix. Peak force microscopy studies revealed that the scaffolds had high mechanical strength and the Young’s Modulus increased with conjugation with each protein layer. The scaffolds also displayed biodegradability. Furthermore, the scaffolds were found to promote cell proliferation and encouraged neurite outgrowths. Although cell proliferation was relatively lower for the PANI bound scaffolds, compared to scaffolds without PANI, our results indicated that the biological components of the scaffold overall aided in cell growth. Additionally, cyclic voltammetry conducted showed that PANI bound scaffolds displayed electrochemical properties. Thus, we have created supramolecular composite scaffolds that may be have applications in neural tissue engineering.

## Figures and Tables

**Table 1:** Youngs Modulus values of constructs after consecutive incorporation of each layer.

Construct	Young’s Modulus (GPa)
Self-Assembled nanoribbons	0.757
Nanoribbons bound to Laminin	1.805
Nanoribbons bound to Laminin-Artemin-Collagen	2.985
Nanoribbons bound to Laminin-Artemin-Collagen-PANI	5.522
